# A Population-Based Systematic Clinical Analysis With a Single-Center Case Series of Patients With Pulmonary Large Cell Neuroendocrine Carcinoma

**DOI:** 10.3389/fendo.2021.759915

**Published:** 2021-12-03

**Authors:** Xu Sun, Yijun Wu, Jing Shen, Chang Han, Kai Kang, Zhikai Liu, Fuquan Zhang

**Affiliations:** ^1^ Department of Radiation Oncology, Peking Union Medical College Hospital, Chinese Academy of Medical Sciences & Peking Union Medical College, Beijing, China; ^2^ Department of Endocrinology, Peking Union Medical College Hospital, Chinese Academy of Medical Sciences & Peking Union Medical College, Beijing, China

**Keywords:** pulmonary large cell neuroendocrine carcinoma, incidence, mortality, survival, treatment

## Abstract

**Background and Objectives:**

This study aims to conduct an updated systematic analysis of patients with pulmonary large cell neuroendocrine carcinoma (PLCNC) in recent decades, concerning incidence and mortality trends, demographics, treatments, survival and death causes.

**Methods:**

Patients who were diagnosed with PLCNC at the Peking Union Medical College Hospital (PUMCH) between 2000 to 2020 were retrospectively analyzed. The population-based Surveillance, Epidemiology, and End Results (SEER) database were also retrieved. Frequencies and average annual age-adjusted rates (AAR) of PLCNC patients were calculated and analyzed by Joint-point regression. Univariate and multivariate Cox regression were used for identifying prognostic factors. Predictive nomograms for overall survival (OS) and cancer-specific survival (CSS) were developed and then validated by calculating C-index values and drawing calibration curves. Survival curves were plotted using the Kaplan-Meier method and compared by log-rank test. Causes of death were also analyzed by time latency.

**Results:**

A total of 56 PLCNC patients of the PUMCH cohort were included. Additionally, the PLCNC patients in the SEER database were also identified from different subsets. The AAR from 2001 to 2017 were 3.21 (95%CI: 3.12-3.30) per million. Its incidence and mortality rates in PLCNC patients increased at first but seemed to decline in recent years. Besides TNM stage and treatments, older age and male gender were independently associated with poorer survival, while marital status only affected CSS other than OS. The nomograms for OS and CSS presented great predictive ability and calibration performance. Surgery gave significantly more survival benefits to PLCNC patients, and chemotherapy might add survival benefits to stage II-IV. However, radiation therapy seemed to only improve stage III patients’ survival.

**Conclusions:**

This study supported some previous studies in terms of incidence, survival, and treatment options. The mortality rates seemed to decline recently, after an earlier increase. Among PLCNC patients, most of the deaths occurred within the first five years, while other non-PLCNC diseases increased after that. Thus, careful management and follow-up of other comorbidities are of equal importance. Our study may partly solve the dilemma caused by PLCNC’s rarity and inspire more insights in future researches.

## Introduction

Lung cancer remains one of the most lethal cancers worldwide and causes countless deaths. Pulmonary large cell neuroendocrine carcinoma (PLCNC), one special rare subtype of non-small cell lung cancer (NSCLC), accounts for approximately 3% of pulmonary carcinomas throughout America (1). Demonstrating differences from other NSCLC subtypes, such as lung adenocarcinoma (ADC) and squamous cell carcinoma (SCC), PLCNC demonstrated disappointing survival outcomes as small cell lung cancer (SCLC) (1, 2), both of which were classified as pulmonary neuroendocrine neoplasms in the 2015 World Health Organization tumor classification (3). Currently, descriptions of PLCNC with regard to clinicopathological manifestations, treatments, and outcomes have not been reported clearly and adequately because of its rarity. Meanwhile, its incidence seems to keep increasing over recent years (4). Therefore, systematically analyzing PLCNC patients could provide reference values for understanding the rare disease.

Diagnosis of PLCNC mainly depends on autopsy or postoperative histology, and small-size PLCNC may be hard to distinguish from other histological types of NSCLC. Thus, most previous studies have included retrospective cohorts and randomized controlled multicenter clinical trials have been very few, which limited the disease’s advances in terms of diagnosis and therapy. None of the standard guidelines have been proposed yet. For primary early-stage PLCNC, surgical resection is first considered like other NSCLC subtypes. However, there were no specific regimens with definite survival benefits that have been fully recommended for locally advanced and advanced PLCNC cases (5–7).

Compared with ADC and SCC, PLCNC presented more malignant behaviors and invasive ability. High local recurrence and metastasis rates were observed in PLCNC patients, which resulted in poor survival outcomes (8, 9). Some studies have reported variable 5-year survival rates but there have been a limited number of cases (8–12). There is still a lack of large cohorts of PLCNC patients showing its long-term survival outcome.

In the present study, a case series of PLCNC patients at our hospital were collected to analyze clinical manifestations and survival outcomes. The Surveillance, Epidemiology, and End Results (SEER) program established by the National Cancer Institute in the United States was searched and analyzed to systematically investigate its incidence, mortality, clinical characteristics, treatments, survival outcomes, and death causes, aiming to help address shortages of corresponding studies.

## Materials and Methods

### Patient Selection

Patients who were diagnosed with PLCNC at the Peking Union Medical College Hospital (PUMCH) from January 2000 to January 2020 were retrospectively collected. The diagnosis of PLCNC was confirmed by histology from aspiration biopsy or bronchoscopy or surgical specimen, which fulfilled the following criteria: 1) neuroendocrine morphology; 2) high ratio of cell division; 3) necrosis; 4) cytological characteristics of NSCLC; 5) immunohistochemistry results: at least one positive biomarker of synaptophysin (Syn), chromogranin A (chromogranin A, CgA) and CD56, or neuroendocrine granules could be found under the electron microscope. Additionally, the immunohistochemical biomarkers also involved cell keratin-7 (CK7), Ki-67, and thyroid transcription factor-1 (TTF-1). The serum biomarker included neuron-specific enolase (NSE) and cytokeratin 19 fragments (Cyfra 21-1). TNM staging was based on AJCC, the eightth edition. Operations included lobectomy, segment and wedge resection with systematical or sublevel lymph node resection, but did not include biopsy surgery. The adjuvant chemotherapy regimens included pemetrexed, docetaxel, gemcitabine, or paclitaxel combined with platinum 

The public SEER database was retrieved for PLCNC, using SEER†Stat 8.3.8 software (https://seer.cancer.gov/seerstat/) with the initial strategy of “Site recode B ICD-O-3/WHO 2008 = Lung and Bronchus AND Site and Morphology ICD-O-3 His/behav = 8013/3: Large cell neuroendocrine carcinoma”. In the SEER program, there are a series of subsets, including incidence, mortality, custom, and other databases. Corresponding analyses were conducted using these databases, respectively. Moreover, two kinds of information sources in the database were applied, and one includes nine registries (Atlanta, Connecticut, Detroit, Hawaii, Iowa, New Mexico, San Francisco–Oakland, Seattle–Puget Sound, and Utah) while the other does eighteen registries (Alaska, Connecticut, Detroit, Atlanta, Greater Georgia, Rural Georgia, San Francisco-Oakland, San Jose-Monterey, California, Hawaii, Iowa, Kentucky, Los Angeles, Louisiana, New Mexico, New Jersey, Seattle–Puget Sound and Utah). Patients from the SEER database were enrolled who met the following criteria: 1) primary PLCNC confirmed by histology; 2) age of diagnosis ≥18 years old; and 3) year of diagnosis: 2001-2016. The exclusion criteria included: 1) not first primary tumor; 2) indefinite follow-up or vital status; 3) died within one month after diagnosis; and 4) unspecific critical demographic or treatment information. The study was conducted in accordance with the Declaration of Helsinki and all patients in the SEER database were anonymous. All patients from our center signed informed consent, and ethical approval was given by the Ethics Committee of Peking Union Medical College Hospital.

### Incidence and Death

Despite PLCNC’s rarity, the SEER database can be used to accurately investigate the incidence and death trends in recent decades. In this subtopic, using the Incidence-SEER Research Data with 18 Registries (November 2019 Submitted) and Incidence-Based Mortality-SEER Research Data with 9 Registries (November 2019 Submitted), we calculated the frequencies, incidence, and death rates of PLCNC and analyzed its trends by Join-point regression (https://surveillance.cancer.gov/joinpoint/), respectively. Using SEER†Stat software, the Join-point analysis finds the best fitting piecewise continuous log-linear model and identified the minimal number of join points by permutation test. The incidence rates at each age range were also plotted and analyzed.

### Clinical Parameters in the SEER Database

Descriptions of clinical demographics and death causes were based on the SEER 18 Regs Custom Data (with additional treatment fields; November 2018 Submitted, 1975-2016). The identified variables from the SEER database were as follows: age of diagnosis, race (white, black, others [American Indian/Alaska Native, Asian or Pacific Islander], gender, year of diagnosis (2001-2016), primary site (upper lobe, middle lobe, lower lobe and others/unknown), grade, laterality, tumor extent (localized, regional and distant), AJCC sixth and seventh edition staging, surgical resection (none, wedge resection, segmentectomy, lobectomy, and other surgery), lymph node resection (none, 1-3 regional nodes removed, 4 or more regional nodes removed, biopsy or aspiration of regional nodes), chemotherapy, radiotherapy, distant metastasis (bone, liver, brain, and lung) and marital status (married, single [never married, widowed, divorced/separated]).

### Statistical Analysis

In the study, all statistical analyses were carried out using R software 3.6.3 (https://www.r-project.org) and IBM SPSS 26.0 except those of incidence and death rates, which were performed by the SEER†Stat 8.3.8 for Join-point regression analyses. P value <0.05 was considered statistically significant. Univariate and multivariate Cox proportional hazard models were constructed to identify possible prognostic factors for overall survival (OS) and cancer-specific survival (CSS). Variables with P<0.05 in the univariate Cox regression were involved for multivariate analysis (LR forward). Survival curves were drawn using the Kaplan-Meier method and compared by log-rank test.

Furthermore, nomograms were developed to estimate PLCNC patients’ 1-, 3- and 5-year OS and CSS. Before modeling, patients were randomly split into training and validation cohorts by a ratio of 7:3., for which C-index values were calculated to evaluate nomograms’ predictive performance, respectively. Additionally, calibration plots had also been validated internally and externally.

## Results

### PLCNC Patients of the PUMCH Cohort

**Figure 1:**
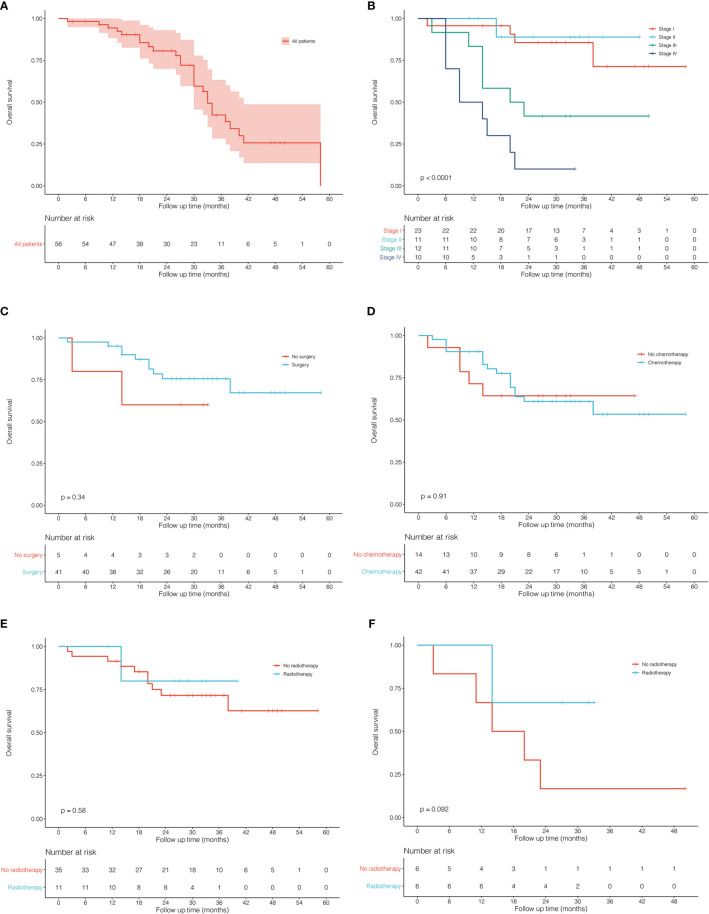
Kaplan–Meier curves for overall survival in patients with pulmonary large cell neuroendocrine carcinoma from Peking Union Medical College Hospital. **(A)** all 56 patients. **(B)** comparison between different stages. **(C)** surgery *vs* no surgery in stage I-III patients. **(D)** chemotherapy *vs* no chemotherapy in all patients. **(E)** radiotherapy *vs* no radiotherapy in all patients. **(F)** radiotherapy *vs* no radiotherapy in stage III patients.

A total of 56 PLCNC patients from PUMCH were enrolled, including 48 male and 8 female patients (**Table 1**). The median age was 63 (IQR: 60-69) years old. Most of the patients (85.7%, 48/56) had a smoking history. Few patients demonstrated abnormal serum levels of NSE (21.4%, 12/56) and Cyfra21-1 (10.7%, 6/56). The immunohistochemistry results showed high positive rates of CgA (60.7%, 34/56), Syn (78.6%, 44/56), CD56 (67.9%, 38/56), CK7 (75.0%, 42/56) and TTF-1 (62.5%, 35/56) with a severe median Ki-67 index of 80% (IQR: 70%-85%). Of all patients, 45 (80.4%) received surgical resection (lobectomy: 38 cases; wedge resection: 5 cases; segment resection: 2 cases). Most of the patients (76.8%, 43/56) received adjuvant chemotherapy, and 13 (23.2%) patients underwent radiation therapy. **Figure 1** showed the survival outcome of the PUMCH cohort. The median follow-up time was 26 (IQR: 14-33) months. The 1-year, 2-year, and 3-year OS for all PLCNC patients of the PUMCH cohort were 83.9%, 53.6%, and 19.6%, respectively. Stage I and II patients had significantly better survival than stage III and IV (**Figure 1B**). Stage I-III patients seemed to demonstrate better OS after surgery compared with those not receiving any surgery, though there was no significance (**Figure 1C**). In our cohort, no significant differences were observed between patients who underwent adjuvant therapy (chemotherapy in **Figure 1D**; radiotherapy in **Figure 1E**) and those who did not. However, radiotherapy seemed to add survival benefits to stage III patients (P=0.092; **Figure 1F**).

**Table 1:** Clinical characteristics of patients with pulmonary large cell neuroendocrine carcinoma from the PUMCH cohort.

Characteristic	Count	%
Age, median (year)	–	63 (60-69)
Gender		
Male	48	85.7
Female	8	14.3
Smoking history		
Yes	48	85.7
No	8	14.3
Stage		
I	23	41.1
II	12	21.4
III	12	21.4
IV	9	16.1
NSE		
Abonormal	12	21.4
Normal	38	67.9
Unknown	6	10.7
Cyfra21-1		
Abonormal	6	10.7
Normal	44	78.6
Unknown	6	10.7
CgA		
Positive	34	60.7
Negative	20	35.7
Unknown	2	3.6
Syn		
Positive	44	78.6
Negative	11	19.6
Unknown	1	1.8
Ki-67 index, median (%)	–	80 (70-85)
CD56		
Positive	38	67.9
Negative	14	25
Unknown	4	7.1
CK7		
Positive	42	75
Negative	11	19.6
Unknown	3	5.4
TTF-1		
Positive	35	62.5
Negative	18	32.1
Unknown	3	5.4
Surgery		
Yes	45	80.4
No	11	19.6
Chemotherapy		
Yes	43	76.8
No	13	23.2
Radiotherapy		
Yes	13	23.2
No	43	76.8

NSE, neuron-specific enolase; Cyfra21-1, cytokeratin 19 fragments; CgA, chromogranin A; Syn, synaptophysin; CK-7, cell keratin-7; TTF-1, thyroid transcription factor-1.

### Incidence and Mortality Rate Analysis

**Figure 2:**
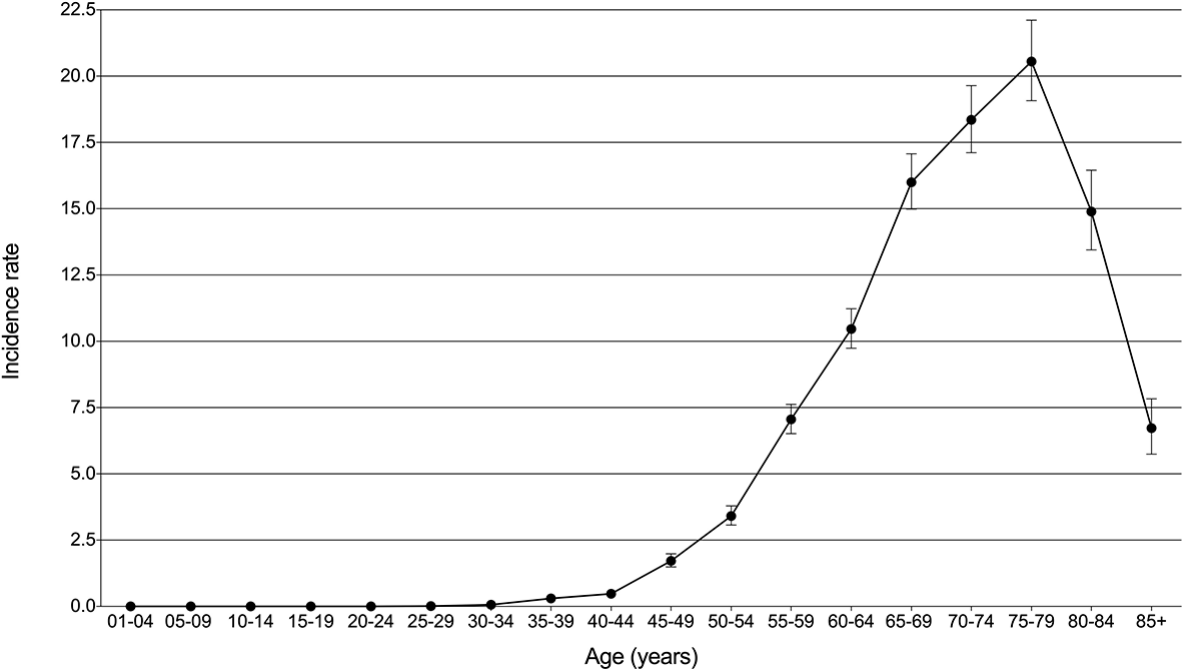
Incidence rates (per million) with 95% CI over age for pulmonary large cell neuroendocrine carcinoma.

Using the subsets of SEER-based databases, incidence and mortality statistics were performed to analyze their change trends. A total of 5068 patients diagnosed with PLCNC between 2001 and 2017 were identified with an average annual age-adjusted rate (AAR) of 3.21 (95%CI: 3.12-3.30) per 1,000,000. **Table 2** presents the incidence rates among population subgroups. Males (3.91, 95%CI: 3.76-4.06) demonstrated a little higher AAR than females (2.67, 95%CI: 2.56-2.78). White (3.35, 95%CI: 3.24-3.45) or black people (3.88, 95%CI: 3.57-4.21) were more likely to suffer PLCNC than American Indian/Alaska Native (1.41, 0.82-2.23) and Asian or Pacific Islander (1.36, 1.17-1.57). Regarding tumor locations, PLCNC was more commonly observed in the right lung (1.78, 95%CI: 1.71-1.84) and upper lobe (1.71, 95%CI: 1.64-1.77). As shown in **Figure 2**, higher rates were associated with an increase in age after 35 years old, and the 75-79 group reached the highest (20.55, 95%CI: 19.08-22.11).

**Table 2:** Population-based frequencies and age-adjusted incidence rates for patients with pulmonary large cell neuroendocrine carcinoma from the Incidence-SEER Research Data with 18 Registries (November 2019 Submitted) between 2001 and 2017.

Characteristic	Count	Rate	SE	95%CI
Total	5068	3.21	0.05	3.12-3.30
Gender				
Male	2783	3.91	0.08	3.76-4.06
Female	2285	2.67	0.06	2.56-2.78
Race				
White	4233	3.35	0.05	3.24-3.45
Black	620	3.88	0.16	3.57-4.21
American Indian/Alaska Native	19	1.41	0.34	0.82-2.23
Asian or Pacific Islander	191	1.36	0.10	1.17-1.57
Unknown	5	–	–	–
Laterality				
Left	2006	1.27	0.03	1.22-1.33
Right	2803	1.78	0.03	1.71-1.84
Unknown	259	–	–	–
Primary site				
Upper lobe	2707	1.71	0.03	1.64-1.77
Middle lobe	234	0.15	0.01	0.13-0.17
Lower lobe	1192	0.77	0.02	0.72-0.81
Unknown	935	–	–	–

**Figure 3:**
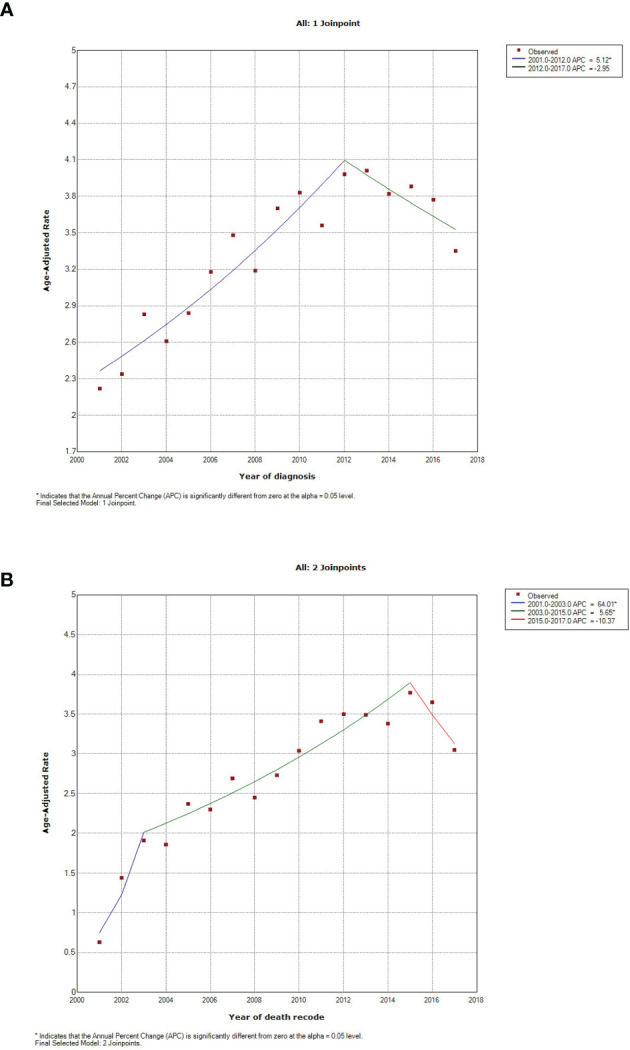
Incidence and mortality trends in patients with pulmonary large cell neuroendocrine carcinoma between 2001 and 2017. **(A)** incidence, using Incidence-SEER Research Data with 18 Registries (November 2019 Submitted); **(B)** mortality, using Incidence-Based Mortality-SEER Research Data with nine Registries (November 2019 Submitted).

Through Join-point regression analyses, significant changes were observed in the incidence and death trends over years per 1,000,000 since 2001 (**Figure 3**). For the incidence, there was one joinpoint identified to divide the time into two periods: 2001-2012 and 2012-2017. The first period showed one significant increase of 5.12% per year (P<0.001), followed by an insignificant decline of -2.95% per year (P=0.099) for the second period (**Figure 3A**). The mortality rates constantly rose until 2015, and were cut into two significant increasing periods and one decreasing period by two join points: 2001-2003 (64.01% per year, P=0.032), 2003-2015 (5.65% per year, P<0.001), and 2015-2017 (-10.37% per year, P=0.235), respectively (**Figure 3B**).

### Prognostic Factor Selection and Nomogram Development

In this section, a total of 4255 patients diagnosed with PLCNC were identified from the SEER 18 Regs Custom Data (with additional treatment fields; November 2018 Submitted, 1975-2016). Using univariate (**Table 3**) and multivariate (**Table 4**) Cox regression analyses, the possible prognostic factors for the OS and CSS of PLCNC patients have been identified. Besides conventional TNM stage, higher age (OS: HR=1.02, 95%CI: 1.01-1.02, P<0.001; CSS: HR=1.02, 95%CI: 1.02-1.03, P<0.001), and male gender (female *vs*. male, OS: HR=0.84, 95%CI: 0.79-0.90, P<0.001; CSS: HR=0.82, 95%CI: 0.76-0.89, P<0.001) were significantly associated with poorer OS and CSS. Compared with those who underwent no surgical resections, patients receiving pulmonary surgery could demonstrate a better prognosis. However, chemotherapy was only significant for OS, while marital status merely influenced CSS.

**Table 3:** Univariate Cox regression analyses for patients with pulmonary large cell neuroendocrine carcinoma using SEER 18 Regs Custom Data (with additional treatment fields; November 2018 Submitted).

Characteristic		Overall survival (N = 4255)				Cancer-specific survival (N = 3529)			
		N (%)	HR	95% CI	P value	N (%)	HR	95% CI	P value
Age, median (IQR)		67 (59-74)	1.02	1.01-1.02	<0.001	66 (59-74)	1.02	1.01-1.02	<0.001
Sex	Male	2292 (53.9)	Ref	–	–	1907 (54)	Ref	–	–
	Female	1963 (46.1)	0.83	0.77-0.89	<0.001	1622 (46)	0.82	0.76-0.88	<0.001
Race	White	3553 (83.5)	Ref	–	–	2936 (83.2)	Ref	–	–
	Black	519 (12.2)	1.02	0.92-1.14	0.665	443 (12.6)	1.01	0.90-1.14	0.807
	Others	180 (4.2)	1.1	0.94-1.30	0.241	147 (4.2)	1.12	0.93-1.34	0.236
	Unknown	3 (0.1)	–	–	–	3 (0.1)	–	–	–
Diagnosis year	2001-2004	700 (16.5)	Ref	–	–	560 (15.9)	Ref	–	–
	2005-2008	935 (22)	1.03	0.92-1.14	0.631	756 (21.4)	0.99	0.88-1.11	0.833
	2009-2012	1228 (28.9)	1.08	0.98-1.20	0.122	995 (28.2)	0.94	0.84-1.05	0.286
	2013-2016	1392 (32.7)	1.16	1.05-1.29	0.005	1218 (34.5)	0.91	0.81-1.02	0.104
Primary site	Upper lobe	2312 (54.3)	Ref	–	–	1942 (55)	Ref	–	–
	Middle lobe	198 (4.7)	1.09	0.93-1.29	0.269	158 (4.5)	1.13	0.94-1.36	0.833
	Lower lobe	1017 (23.9)	1.1	1.01-1.19	0.033	843 (23.9)	1.09	0.99-1.20	0.286
	Others/Unknown	728 (17.1)	2	1.82-2.18	<0.001	586 (16.6)	2.03	1.84-2.25	0.104
Laterality	Left	1687 (39.6)	Ref	–	–	1404 (39.8)	Ref	–	–
	Right	2367 (55.6)	1.01	0.94-1.08	0.824	1988 (56.3)	1.01	0.93-1.09	0.888
	Unknown	201 (4.7)	1.73	1.48-2.02	<0.001	137 (3.9)	1.65	1.37-2.00	<0.001
Grade	I+II	86 (2)	Ref	–	–	67 (1.9)	Ref	–	–
	III	1530 (36)	1.17	0.90-1.51	0.238	1271 (36)	1.19	0.87-1.62	0.27
	IV	479 (11.3)	1.28	0.98-1.68	0.075	395 (11.2)	1.34	0.97-1.85	0.078
	Unknown	2160 (50.8)	1.91	1.48-2.47	<0.001	1796 (50.9)	1.99	1.47-2.71	<0.001
Tumor extent	Localized	893 (21)	Ref	–	–	682 (19.3)	Ref	–	–
	Regional	1119 (26.3)	1.62	1.46-1.80	<0.001	923 (26.2)	1.88	1.65-2.14	<0.001
	Distant	1867 (43.9)	4.41	3.99-2.86	<0.001	1585 (44.9)	5.26	4.66-5.93	<0.001
	Unknown	376 (8.8)	2.49	2.06-3.01	<0.001	339 (9.6)	2.39	1.91-3.00	<0.001
Stage, 7ed AJCC	IA	281 (6.6)	Ref	–	–	224 (6.3)	Ref	–	–
	IB	157 (3.7)	1.08	0.80-1.45	0.614	134 (3.8)	1.24	0.85-1.81	0.265
	IIA	106 (2.5)	1.93	1.44-2.59	<0.001	93 (2.6)	2.66	1.86-3.80	<0.001
	IIB	95 (2.2)	1.76	1.29-2.40	<0.001	77 (2.2)	2.25	1.52-3.32	<0.001
	IIIA	239 (5.6)	2.52	2.00-3.17	<0.001	206 (5.8)	3.67	2.75-4.91	<0.001
	IIIB	109 (2.6)	3.59	2.74-4.70	<0.001	97 (2.7)	5.4	3.90-7.47	<0.001
	IV	958 (22.5)	6.16	5.09-7.45	<0.001	797 (22.6)	9.13	7.09-11.77	<0.001
	Unknown	2310 (54.3)	2.67	2.22-3.21	<0.001	1901 (53.9)	4.33	3.39-5.55	<0.001
T stage, 7ed AJCC	T1	484 (11.4)	Ref	–	–	390 (11.1)	Ref	–	–
	T2	553 (13)	1.58	1.36-1.84	<0.001	463 (13.1)	1.73	1.44-2.06	<0.001
	T3	385 (9)	2.18	1.86-2.55	<0.001	334 (9.5)	2.57	2.14-3.09	<0.001
	T4	381 (9)	3.15	2.69-3.68	<0.001	331 (9.4)	3.71	3.09-4.44	<0.001
	TX	182 (4.3)	2.96	2.45-3.59	<0.001	141 (4)	3.42	2.73-4.29	<0.001
	Unknown	2270 (53.3)	1.58	1.40-1.79	<0.001	1870 (53)	2.05	1.77-2.38	<0.001
N stage, 7ed AJCC	N0	823 (19.3)	Ref	–	–	674 (19.1)	Ref	–	–
	N1	189 (4.4)	1.8	1.50-2.15	<0.001	170 (4.8)	2.08	1.71-2.54	<0.001
	N2	651 (15.3)	2.56	2.27-2.89	<0.001	544 (15.4)	3.02	2.63-3.48	<0.001
	N3	264 (6.2)	3.21	2.75-3.75	<0.001	230 (6.5)	3.86	3.24-4.59	<0.001
	NX	74 (1.7)	3.26	2.53-4.19	<0.001	52 (1.5)	3.51	2.59-4.75	<0.001
	Unknown	2254 (53)	1.52	1.38-1.68	<0.001	1859 (52.7)	1.96	1.74-2.21	<0.001
M stage, 7ed AJCC	M0	1043 (24.5)	Ref	–	–	873 (24.7)	Ref	–	–
	M1a	129 (3)	3	2.47-3.66	<0.001	111 (3.1)	3.31	2.67-4.12	<0.001
	M1b	819 (19.2)	3.57	3.21-3.97	<0.001	678 (19.2)	4.01	3.55-4.53	<0.001
	Unknown	2264 (53.2)	1.52	1.39-1.66	<0.001	1867 (52.9)	1.87	1.69-2.08	<0.001
Stage, 6ed AJCC	IA	498 (11.7)	Ref	–	–	374 (10.6)	Ref	–	–
	IB	421 (9.9)	1.06	0.89-1.26	0.492	346 (9.8)	1.21	0.98-1.51	0.083
	IIA	64 (1.5)	1.43	1.04-1.97	0.027	55 (1.6)	1.79	1.24-2.57	0.002
	IIB	146 (3.4)	1.82	1.46-2.26	<0.001	120 (3.4)	2.26	1.74-2.94	<0.001
	IIIA	301 (7.1)	2.08	1.76-2.47	<0.001	249 (7.1)	2.69	2.18-3.31	<0.001
	IIIB	409 (9.6)	2.98	2.55-3.49	<0.001	350 (9.9)	4.01	3.30-4.86	<0.001
	IV	1481 (34.8)	5.16	4.53-5.88	<0.001	1245 (35.3)	6.82	5.77-8.07	<0.001
	Unknown	935 (22)	2.4	2.09-2.77	<0.001	790 (22.4)	3.32	2.78-3.97	<0.001
T stage, 6ed AJCC	T1	833 (19.6)	Ref	–	–	647 (18.3)	Ref	–	–
	T2	1110 (26.1)	1.36	1.22-1.51	<0.001	916 (26)	1.46	1.28-1.65	<0.001
	T3	172 (4)	1.73	1.44-2.07	<0.001	152 (4.3)	2.02	1.65-2.47	<0.001
	T4	953 (22.4)	2.81	2.52-3.12	<0.001	834 (23.6)	3.27	2.89-3.70	<0.001
	TX	310 (7.3)	2.45	2.12-2.82	<0.001	244 (6.9)	2.78	2.36-3.28	<0.001
	Unknown	877 (20.6)	1.56	1.39-1.75	<0.001	736 (20.9)	1.84	1.61-2.11	<0.001
N stage, 6ed AJCC	N0	1442 (33.9)	Ref	–	–	1142 (32.4)	Ref	–	–
	N1	331 (7.8)	1.57	1.37-1.79	<0.001	292 (8.3)	1.76	1.52-2.05	<0.001
	N2	1073 (25.2)	2.38	2.17-2.60	<0.001	902 (25.6)	2.75	2.48-3.05	<0.001
	N3	421 (9.9)	3.2	2.84-3.60	<0.001	369 (10.5)	3.78	3.31-4.31	<0.001
	NX	146 (3.4)	2.82	2.36-3.37	<0.001	110 (3.1)	3.3	2.67-4.06	<0.001
	Unknown	842 (19.8)	1.53	1.38-1.70	<0.001	714 (20.2)	1.82	1.62-2.06	<0.001
M stage, 6ed AJCC	M0	1865 (43.8)	Ref	–	–	1512 (42.8)	Ref	–	–
	M1	1481 (34.8)	3.28	3.03-3.55	<0.001	1245 (35.3)	3.53	3.23-3.86	<0.001
	MX	67 (1.6)	2.01	1.55-2.61	<0.001	58 (1.6)	2.14	1.61-2.83	<0.001
	Unknown	842 (19.8)	1.51	1.37-1.67	<0.001	714 (20.2)	1.72	1.53-1.93	<0.001
Metastasis	Bone	373 (8.8)	1.02	0.99-1.06	0.236	313 (8.9)	3.08	2.69-3.53	<0.001
	Liver	364 (8.6)	1.03	0.99-1.07	0.138	356 (10.1)	1.02	0.98-1.06	0.352
	Brain	408 (9.6)	1.02	0.98-1.06	0.352	298 (8.4)	1.03	0.99-1.07	0.138
	Lung	237 (5.6)	1.01	0.97-1.04	0.795	198 (5.6)	1.01	0.97-1.04	0.795
Surgery	None	2559 (60.1)	Ref	–	–	2181 (61.8)	Ref	–	–
	Wedge	327 (7.7)	0.37	0.32-0.42	<0.001	252 (7.1)	0.33	0.28-0.39	<0.001
	Segmentectomy	63 (1.5)	0.34	0.25-0.46	<0.001	51 (1.4)	0.31	0.22-0.45	<0.001
	Lobectomy	1110 (26.1)	0.25	0.22-0.27	<0.001	894 (25.3)	0.21	0.19-0.24	<0.001
	Pneumonectomy	68 (1.6)	0.42	0.32-0.55	<0.001	52 (1.5)	0.39	0.28-0.54	<0.001
	Others	128 (3)	0.39	0.32-0.548	<0.001	99 (2.8)	0.35	0.27-0.45	<0.001
Lymph node resection	None	2178 (51.2)	Ref	–	–	1824 (51.7)	Ref	–	–
	1-3 regional nodes removed	349 (8.2)	0.42	0.37-0.48	<0.001	273 (7.7)	0.39	0.34-0.46	<0.001
	≥4 regional nodes removed	1020 (24)	0.29	0.26-0.32	<0.001	847 (24)	0.25	0.22-0.28	<0.001
	Biopsy or aspiration	261 (6.1)	0.86	0.74-0.99	0.031	223 (6.3)	0.86	0.74-1.01	0.058
	Unknown	447 (10.5)	0.52	0.46-0.58	<0.001	362 (10.3)	0.53	0.47-0.60	<0.001
Marital status	Married	2275 (53.5)	Ref	–	–	1902 (53.9)	Ref	–	–
	Single (never married)	583 (13.7)	1.15	1.04-1.28	0.006	483 (13.7)	1.12	0.99-1.25	0.065
	Widowed	602 (14.1)	1.18	1.07-1.30	0.001	482 (13.7)	1.2	1.06-1.33	0.002
	Divorced/separated	616 (14.5)	0.99	0.90-1.10	0.902	515 (14.6)	0.99	0.88-1.11	0.799
	Unknown	179 (4.2)	0.95	0.80-1.14	0.583	147 (4.2)	0.92	0.75-1.13	0.922
Chemotherapy	Yes *vs*. No	2276 (53.5)	1.12	1.04-1.19	0.002	1958 (55.5)	1.15	1.39-1.63	<0.001
Radiation	Yes *vs*. No	1693 (39.8)	1.43	1.34-1.54	<0.001	1447 (41)	1.51	1.06-1.24	0.001

**Table 4:** Multivariate Cox regression analyses for overall survival and cancer-specific survival in patients with pulmonary large cell neuroendocrine carcinoma using SEER 18 Regs Custom Data (with additional treatment fields; November 2018 Submitted).

Characteristic		Overall survival			Characteristic		Cancer-specific survival		
		HR	95% CI	P value			HR	95% CI	P value
Age	–	1.02	1.01-1.02	<0.001	Age	–	1.02	1.02-1.03	<0.001
Sex	Male	Ref	–	–	Sex	Male	Ref	–	–
	Female	0.84	0.79-0.90	<0.001		Female	0.82	0.76-0.89	<0.001
Tumor extent	Localized	Ref	–	–	Tumor extent	Localized	Ref	–	–
	Regional	1.25	1.07-1.47	0.004		Regional	1.13	0.93-1.36	0.218
	Distant	1.77	1.48-2.13	<0.001		Distant	1.53	1.24-1.89	<0.001
	Unknown	1.46	1.16-1.85	0.001		Unknown	0.97	0.74-1.26	0.794
Stage, 7ed AJCC	IA	Ref	–	–	Stage, 7ed AJCC	IA	Ref	–	–
	IB	1.16	0.80-1.68	0.426		IB	1.28	0.80-2.05	0.298
	IIA	1.88	1.29-2.75	0.001		IIA	2.26	1.43-3.57	<0.001
	IIB	1.46	1.00-2.13	0.052		IIB	1.54	0.96-2.46	0.076
	IIIA	1.43	1.06-1.93	0.019		IIIA	1.69	1.16-2.46	0.007
	IIIB	1.15	0.82-1.60	0.416		IIIB	1.39	0.93-2.09	0.106
	IV	1.63	1.26-2.10	<0.001		IV	2.05	1.47-2.87	<0.001
	Unknown	1.42	1.12-1.80	0.003		Unknown	1.96	1.43-2.69	<0.001
N stage, 6ed AJCC	N0	Ref	–	–	N stage, 6ed AJCC	N0	Ref	–	–
	N1	1.15	0.97-1.37	0.116		N1	1.19	0.99-1.44	0.069
	N2	1.29	1.14-1.46	<0.001		N2	1.3	1.14-1.50	<0.001
	N3	1.43	1.24-1.66	<0.001		N3	1.41	1.20-1.65	<0.001
	NX	1.28	1.04-1.59	0.022		NX	1.38	1.08-1.75	0.009
	Unknown	1.95	1.26-3.03	0.003		Unknown	2.36	1.38-4.03	0.002
Surgery	None	Ref	–	–	Surgery	None	Ref	–	–
	Wedge	0.56	0.48-0.65	<0.001		Wedge	0.57	0.47-0.68	<0.001
	Segmentectomy	0.52	0.38-0.71	<0.001		Segmentectomy	0.55	0.39-0.79	0.001
	Lobectomy	0.39	0.35-0.44	<0.001		Lobectomy	0.4	0.34-0.45	<0.001
	Pneumonectomy	0.62	0.47-0.83	0.001		Pneumonectomy	0.63	0.44-0.89	0.009
	Others	0.51	0.41-0.64	<0.001		Others	0.5	0.38-0.64	<0.001
Chemotherapy	Yes *vs*. No	0.62	0.57-0.67	<0.001	Marital status	Married	Ref	–	–
						Single (never married)	1.06	0.94-1.20	0.315
						Widowed	1.21	1.07-1.36	0.002
						Divorced/separated	1.14	1.01-1.28	0.033
						Unknown	1.08	0.87-1.33	0.488

**Figure 4:**
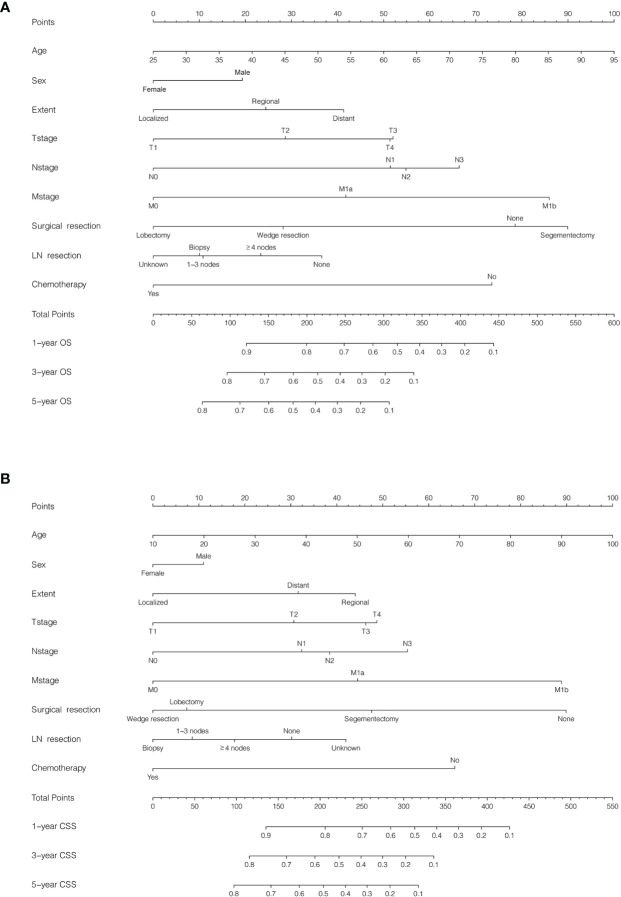
Nomogram for predicting survival in patients with pulmonary large cell neuroendocrine carcinoma using SEER 18 Regs Custom Data (with additional treatment fields; November 2018 Submitted). Patients were randomly split into training and validation cohorts by a ratio of 7:3. **(A)** Overall survival (OS), C-index: 0.775 (95%CI: 0.746-0.804) in the training cohort and 0.729 (95%CI: 0.6760.782) in the validation cohort; **(B)** Cancer-specific survival (CSS), C-index: 0.783 (95%CI: 0.736-0.802) in the training cohort and 0.783 (95%CI: 0.734-0.832) in the validation cohort.

To estimate PLCNC patients’ survival, based on Cox regression results and clinical application, we developed nomograms for succinctly assessing 1-, 3- and 5-year OS (**Figure 4A**) and CSS (**Figure 4B**). In the training cohort, the C-index values of nomogram models for OS and CSS were 0.775 (95%CI: 0.746-0.804) and 0.783 (95%CI: 0.736-0.802), while the values were 0.729 (95%CI: 0.676-0.782) and 0.783 (95%CI: 0.734-0.832) in the validation cohort, respectively. In addition, the calibration curves were also plotted for internal and external validation, which presented great calibration of our nomograms (**Figure S1**).

### Treatment and Survival Analysis of the SEER Cohort

**Figure 5:**
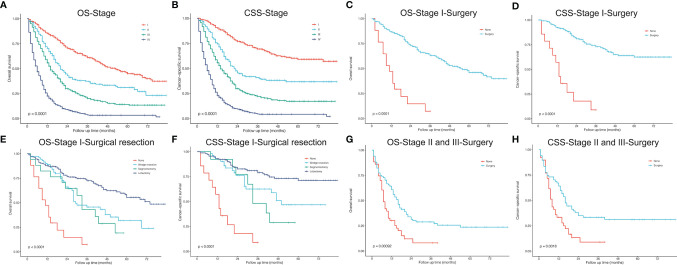
Kaplan–Meier curves. **(A, B)** Stage, AJCC 7th edition; **(C, D)** stage I, surgery *vs*. no surgery; **(E, F)** stage I, surgical resections; **(G, H)** stage II and III, surgery *vs*. no surgery. OS, overall survival; CSS, cancer-specific survival.

To conduct survival comparisons between treatments, patients with complete records of the stage (AJCC, seventh edition) and treatment (surgical resection, chemotherapy, and radiotherapy) were identified, respectively (**Table 5**). Between stages, patients had significantly different OS and CSS (**Figures 5A, B**; P<0.001). Stage I (**Figures 5C, D**, P<0.001), II and III (OS: **Figure 5G**, P<0.001 and CSS: **Figure 5H**, P=0.002) patients undergoing surgery showed significantly better survival outcomes than those who did not receive surgery. Among stage I patients, lobectomy had more survival benefits than wedge resection and segmentectomy (**Figures 5E, F**, P<0.001).

**Table 5:** Distribution of patients with pulmonary large cell neuroendocrine carcinoma who had complete records of TNM stage (AJCC, seventh edition) and treatments using SEER 18 Regs Custom Data (with additional treatment fields; November 2018 Submitted).

Treatment	Total	Stage I	Stage II	Stage III	Stage IV
All patients	1764	437	200	342	785
None	198	17	11	32	138
Surgery					
None	1066	60	43	231	732
Wedge resection	140	91	16	19	14
Segmentectomy	28	22	3	0	3
Lobectomy	460	244	123	68	25
Others	70	20	15	24	11
Radiotherapy					
No	1033	371	151	136	375
Yes	731	66	49	206	410
Chemotherapy					
No	780	351	85	81	263
Yes	984	86	115	261	522
Only surgery	390	294	57	27	12
Wedge resection	81	72	3	3	3
Segmentectomy	19	17	2	0	0
Lobectomy	258	189	43	19	7
Others	32	16	9	5	2
Only radiotherapy	161	27	14	16	104
Only chemotherapy	266	4	6	43	213
Radiotherapy+chemotherapy	441	12	12	140	277
Surgery+radiotherapy	31	13	3	6	9
Surgery+chemotherapy	179	56	77	34	12
Surgery+radiotherapy+chemotherapy	98	14	20	44	20

**Figure 6:**
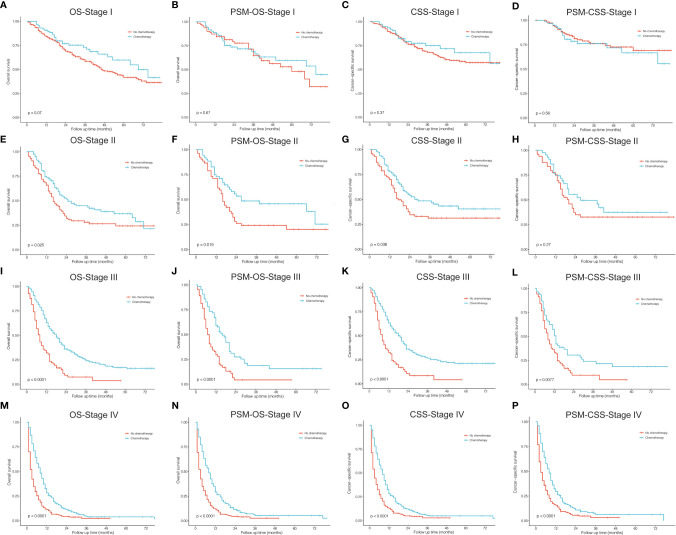
Kaplan–Meier curves for comparisons of chemotherapy vs. no chemotherapy in patients with pulmonary large cell neuroendocrine carcinoma. **(A–D)** Stage I; **(E–H)** Stage II; **(I–L)** Stage III; **(M–P)** Stage IV. OS, overall survival; CSS, cancer-specific survival; PSM, propensity score matching.

**Figure 7:**
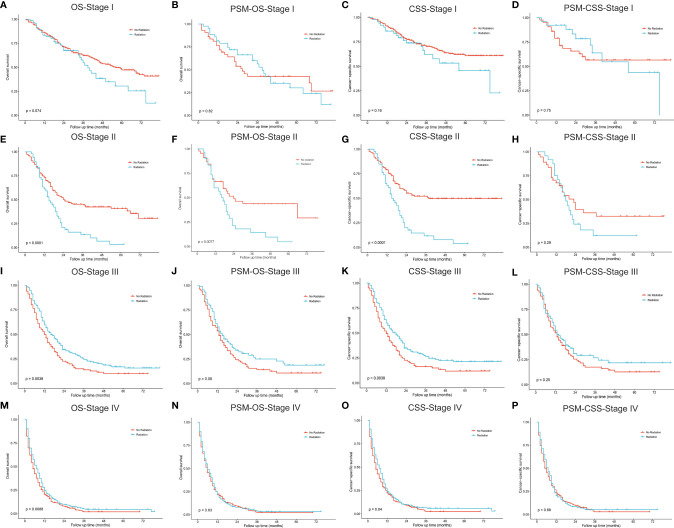
Kaplan–Meier curves for comparisons of radiotherapy vs. no radiotherapy in patients with pulmonary large cell neuroendocrine carcinoma. **(A–D)** Stage I; **(E–H)** Stage II; **(I–L)** Stage III; **(M–P)** Stage IV. OS, overall survival; CSS, cancer-specific survival; PSM, propensity score matching.

**Figures 6**, **7** described the survival comparisons in terms of chemotherapy and radiotherapy, respectively. For each of them, baseline characteristics were adjusted by PSM analysis, including age, race, sex, year of diagnosis, tumor site, laterality, and other treatments. For example, when we compared between performing chemotherapy or not (**Figure 6**), surgical resection and radiation therapy were also matched for balance. OS and CSS were compared separately for patients of each stage. Among stage I cases, chemotherapy did not significantly increase survival benefits of both OS (**Figures 6A, B** **)** and CSS (**Figures 6C, D** **)**, while stage II patients receiving chemotherapy demonstrated significantly OS (**Figure 6E**, P=0.025; PSM: **Figure 6F**, P=0.019) rather than CSS (**Figure 6G**, P=0.036), which presented no significant difference after PSM (**Figure 6H**, P=0.270). For stage III (**Figures 6I–L**) and IV (**Figures 6M–P**), patients receiving chemotherapy obtained significant survival benefits for both OS and CSS, before and after PSM. However, radiotherapy seemed to have no significant increase in benefits for patients of all stages after PSM (**Figure 7**).

### Death Cause

**Figure 8:**
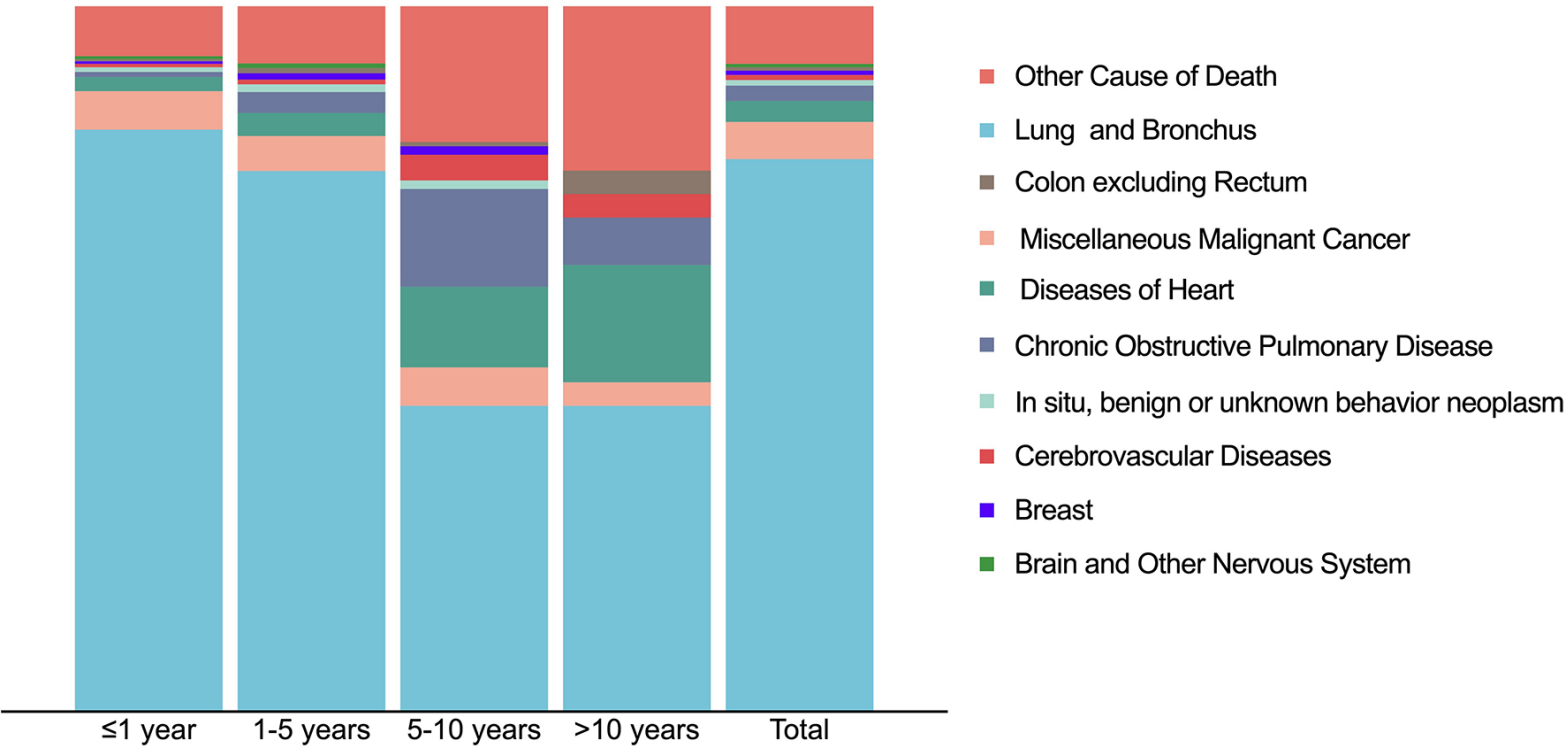
Causes of death distribution in patients with pulmonary large cell neuroendocrine carcinoma using SEER 18 Regs Custom Data (with additional treatment fields; November 2018 Submitted).

The primary disease (“Lung and Bronchus”) was the main cause of death among PLCNC patients (78.3%), especially in the first year (82.5%) and one to five years (76.6%; **Figure 8**). However, it decreased markedly in the five to ten years (43.4%) and more than 10 years’ latency (43.3%). Furthermore, cases who survived more than 5 years would be more likely to suffer chronic obstructive pulmonary disease (5-10 years: 13.9%; >10 years: 6.7%), diseases of the heart (5-10 years: 11.4%; >10 years: 16.7%) and cerebrovascular disease (5-10 years: 3.6%; >10 years: 3.3%). Overall, besides primary disease, the most common death causes included other malignant cancer (5.2%), diseases of the heart (3.0%), and chronic obstructive pulmonary disease (2.2%).

## Discussion

Among lung cancer patients, PLCNC remains a relatively rare type and was reported to account for approximately three percent of all resected pulmonary cancer specimens (1, 13). As one kind of high-grade neuroendocrine tumor, it was noticed and named by Travis et al. in 1991, who first identified a spectrum of tumors that existed between SCLC and typical carcinoid, proposing the disease a new histological category of large-cell neuroendocrine tumor in the lungs (8). Although PLCNC was classified as NSCLC subtypes in the latest version of WHO Classification, it presented genetic and clinical characteristics similar to SCLC. Thus, there are debates about PLCNC (11, 14, 15). In 2015, based on immunohistochemical properties, the WHO association reclassified SCLC and PLCNC as high-grade lung neuroendocrine cancer. The common clinical manifestations were described such as smoking, older age, male, and being easy to metastasis (11, 14–17). The histology check may also be hard to distinguish between PLCNC and SCLC because of similar neuroendocrine characteristics (16). Additionally, due to the low incidence, it remains difficult to perform more advanced research on PLCNC. Therefore, the current problems for PLCNC are connected to it lasting for a long-term period, especially its treatments. In the present study, we retrospectively analyzed 56 PLCNC patients diagnosed at our hospital from 2000 to 2020. Meanwhile, by the application of the population-based SEER database, we systematically analyzed PLCNC’s incidence, mortality, clinical demographics, treatments, survival outcomes and death causes, providing baseline references for the future research on PLCNC.

PLCNC occurred rarely and accounted for approximately 2.4%-3.1% of all lung cancer patients (18, 19). Considering that PLCNC has difficulties in being identified from other lung cancer subtypes in regards to radiology and histology (16, 17), its incidence may have been underestimated. In our study, the PLCNC’s AAR between 2001 and 2017 was 3.21 (95%CI: 3.12-3.30) per million population, similar to other studies (17, 20). Moreover, we observed incidence preponderance in males, whites, and blacks, and right laterality and upper lobe of tumor location (**Table 2**). Gender was reported to be associated with PLCNC but with disparities (4, 17, 21–23). Additionally, incidence rates could increase over age after 35 years old and become higher at 75-79 (20.55 per million; **Figure 2**). In one analysis from two large US commercial claims databases between 2009 and 2014, PLCNC was most commonly found in the 55-64 year-olds (25.1-102.3 per million in different subgroups of the population), in which, however, its incidence might be overestimated because of a small sample of enrolled patients (fewer than one thousand) in the study (21).

The Join-point regression-based trend analysis showed a significant increase in incidence rates by 5.12% per year from 2001 to 2012 (P<0.001), but there was a decrease by -2.95% per year after 2012, though without significance (P=0.099; **Figure 3A**). First of all, the increase may be attributed to the rapid advancement and wide use of radiological technology in recent decades, such as computed tomography (CT) for improving the screening and diagnosis of lung cancer (24). Furthermore, diagnosis and identification for PLCNC have been improved with regards to biomarker tests and histological checks (25). Similarly, the age-adjusted mortality rates of PLCNC have risen since 2001 (P<0.05) but started to decline after 2015 (P=0.235; **Figure 3B**). The increase from 2001 to 2015 may be attributed to rising diagnoses and biopsy cases. Moreover, more deaths caused by PLCNC were confirmed by autopsy. The insignificant decrease in deaths after 2015 can be attributed to early diagnosis and treatment advancement, especially the multidisciplinary treatment (MDT) pattern that involves surgery, chemotherapy, immunotherapy, and radiation therapy, effectively improving its survival outcome and lowering death rates. Nevertheless, in response to the above possible reasons for the trend analyses of incidence and mortality, more evidence is required. Further determining their importance was thus beyond the scope of this study.

The accurate follow-up information in the SEER database made it possible to select valuable prognostic factors for PLCNC. Consistent with previous studies (11, 12, 20, 26–28), male gender and older age were associated with poor survival besides TNM stage and treatments (**Table 4**). Interestingly, we found that marital status could significantly influence CSS instead of OS. Compared with married patients, those with widowed (HR=1.21, 95%CI: 1.07-1.36, P=0.002) and divorced/separated (HR=1.14, 95%CI: 1.01-1.28, P=0.033) status might have worse PLCNC-specific survival outcome. One large study involving 440,000 cancer patients found higher mortality in those who were widowed and unmarried when compared with those who were married (29). This phenomenon, indicated by previous studies may highlight the critical role of partnership or marriage in the management of cancer patients, such as life support, seeking treatment, and help in decision-making from partners (29, 30).

To conveniently and precisely evaluate PLCNC patients’ survival outcome, we developed nomograms for OS and CSS using the following variables: age, sex, tumor extent, TNM stage (AJCC, 7th edition), surgical resection, lymph node resection, and chemotherapy, which were selected based on clinical applications and our Cox regression results. Clinicians and patients worldwide can then use them to succinctly assess survival outcomes (**Figure 4**). The nomogram models were internally and externally validated with great predictive ability (**Figure 4**; all C-index values > 0.7) and calibration performance (**Figure S1**).

Treatment options for PLCNC remain unclear and there is a lack of prospective data and official guidelines. The commonly-used treatment patterns were similar to NSCLC. For stage I-III patients, surgical resection was reported to improve OS significantly, and lobectomy showed the best outcome among stages I and II (27). Similarly, in the SEER cohort, stage I-III patients who received surgery could have a significantly better OS and CSS than those who did not, and lobectomy performed better than sub-lobar resection in stage I cases. In the PUMCH cohort, stage I-III patients who received surgery also demonstrated better survival, though there was no significance because of limited samples (**Figure 1C**). There were not enough patients for comparisons between surgical strategies among stages II and III, and thus, the application of sub-lobar resection requires further evidence from future research.

Among resectable PLCNC cases, one prospective study indicated that adjuvant chemotherapy (cisplatin plus VP-16) was better than surgery alone (31). A series of studies have reported observed improved survival outcomes among cases receiving adjuvant chemotherapy (5, 31–35). Based on the SEER database, our study conducted survival comparisons between receiving chemotherapy and not in each stage and also adjusted baselines by PSM (**Figure 6**). It could be inferred that chemotherapy had no survival benefits to stage I cases (**Figures 6A–D**). For stage II patients, undergoing chemotherapy had a significantly better OS (**Figure 6E**, P=0.025; PSM: **Figure 6F**, P=0.019) but insignificantly different CSS after PSM (before PSM: **Figure 6G**, P=0.036; after PSM: **Figure 6H**, P=0.270). Among stage II (**Figures 6I–L**) and III, chemotherapy could add significantly more benefits to both OS and CSS (**Figures 6M–P**). Due to the lack of regimen information in the SEER database, we cannot carry out specific comparisons to identify the best one. A multi-center retrospective study of 56 cases indicated the regimen for SCLC might perform better than that for NSCLC in terms of disease-free survival (DFS) (35). Nevertheless, there was still a lack of further evidence in chemotherapy regimens for PLCNC. It was reported that surgical resection combined with radiotherapy could not improve OS than surgery alone among early-stage cases, but demonstrated encouraging outcomes in locally advanced and advanced stages (27). In our study, radiotherapy only had significant effects on OS among stage II patients after PSM (P=0.008; **Figure 7**). In our PUMCH cohort, chemotherapy and radiotherapy seemed to have limited benefits for survival outcomes (**Figures 1D, E**). However, radiotherapy might have critical effects on the treatment for stage III cases, though there was no significance because of limited samples (P=0.092; **Figure 1F**). Thus, considering the rarity of the disease, more studies should be conducted to verify the role of chemotherapy and radiotherapy in the treatment of PLCNC.

The leading cause of death among PLCNC patients was the primary tumor. However, as the survival latency was prolonged, these patients were more prone to die from non-PLCNC diseases, including other malignant cancers (5.2%), diseases of the heart (3.0%), and chronic obstructive pulmonary disease (2.2%). For them, careful management and follow-up of other comorbidities may be more critical to survival outcomes.

There are several limitations to this study. First, PLCNC is a rare type of lung cancer, and we used the SEER database to identify a large cohort of patients. However, due to its retrospective nature and lack of some important clinical characteristics (medical history, smoking status, treatment details, etc.), the outcomes require further study to verify the results. Thus, the PUMCH cohort included more detailed information such as immunohistochemistry results and therapy strategies, which are not available in the SEER database. The cohorts from the two sources could complement each other, providing more reference evidence for future studies. Our study first described the meaningful changes of PLCNC’s incidence and mortality using Join-point regression in recent decades, and also systematically analyzed prognostic factors, treatment options, and causes of death. In addition, predictive nomograms for OS and CSS were also developed for applications, though more studies are required for further validation.

## Conclusions

This population-based study conducted an overview analysis of PLCNC patients that aimed to provide useful references for future studies and clinical applications. The AAR for PLCNC from 2001 to 2017 was 3.21 (95%CI: 3.12-3.30) per million. The analyses of incidence and mortality showed a similar trend of rising first and then falling. Besides TNM stage and treatments, older age and male gender were identified to be independent risk factors for OS and CSS, while marital status only affected CSS. Moreover, predictive nomograms for OS and CSS had also been developed with great predictive ability and calibration performance. As for treatments, surgery was recommended for stage I-III, and chemotherapy might add survival benefits to stage II-IV. However, radiation therapy seemed to only improve stage III patients’ OS. Among PLCNC patients, most of the deaths occurred within the first five years, while in other non-PLCNC diseases, death rates increased after that time. Thus, careful management and follow-up of other comorbidities are of equal importance, especially for long-term survivors. Despite requiring further evidence, this study may improve the dilemma of this malignancy’s rarity and inspire further research.

## Data Availability Statement

The datasets presented in this study can be found in online repositories. The names of the repository/repositories and accession number(s) can be found in the article/**Supplementary Material**.

## Ethics Statement

The studies involving human participants were reviewed and approved by the Ethics Committee of Peking Union Medical College Hospital. The patients/participants provided their written informed consent to participate in this study.

## Author Contributions

Conception and design: YW, ZL, and FZ. Provision of study materials or patients: XS, JS, CH, and YW. Collection and assembly of data: JS, KK and ZL. Data analysis and interpretation: XS, JS, and YW. Manuscript writing: All authors. Final approval of manuscript: All authors.

## Funding

This study was funded by the National Key Research and Development Plan, the Ministry of Science and Technology of the People’s Republic of China [grant number 2017YFC1311004]. National Foundation for Education Sciences Planning [grant number BLA200216].

## Conflict of Interest

The authors declare that the research was conducted in the absence of any commercial or financial relationships that could be construed as a potential conflict of interest.

## Publisher’s Note

All claims expressed in this article are solely those of the authors and do not necessarily represent those of their affiliated organizations, or those of the publisher, the editors and the reviewers. Any product that may be evaluated in this article, or claim that may be made by its manufacturer, is not guaranteed or endorsed by the publisher.

## Supplementary Material

Supplementary Figure 1 Calibration curves for nomograms. **(A–C)**: 1-, 3- and 5-year overall survival (OS) validated in the training cohort; **(D–F)**: 1-, 3- and 5-year cancer-specific survival (CSS) validated in the validation cohort; **(G–I)**: 1-, 3- and 5-year OS validated in the training cohort; **(J–L)**: 1-, 3- and 5-year CSS validated in the validation cohort.
